# Detection of PrPres in Genetically Susceptible Fetuses from Sheep with Natural Scrapie

**DOI:** 10.1371/journal.pone.0027525

**Published:** 2011-12-14

**Authors:** María Carmen Garza, Natalia Fernández-Borges, Rosa Bolea, Juan José Badiola, Joaquín Castilla, Eva Monleón

**Affiliations:** 1 Centro de Investigación en Encefalopatías Espongiformes Transmisibles y Enfermedades Emergentes, Universidad de Zaragoza, Zaragoza, Spain; 2 CIC bioGUNE, Parque Tecnológico de Bizkaia, Derio, Spain; 3 IKERBASQUE, Basque Foundation for Science, Bilbao, Spain; 4 Producció Animal, Universitat de Lleida, LLeida, Spain; Creighton University, United States of America

## Abstract

Scrapie is a transmissible spongiform encephalopathy with a wide PrPres dissemination in many non-neural tissues and with high levels of transmissibility within susceptible populations. Mechanisms of transmission are incompletely understood. It is generally assumed that it is horizontally transmitted by direct contact between animals or indirectly through the environment, where scrapie can remain infectious for years. In contrast, *in utero* vertical transmission has never been demonstrated and has rarely been studied. Recently, the use of the protein misfolding cyclic amplification technique (PMCA) has allowed prion detection in various tissues and excretions in which PrPres levels have been undetectable by traditional assays. The main goal of this study was to detect PrPres in fetal tissues and the amniotic fluid from natural scrapie infected ewes using the PMCA technique. Six fetuses from three infected pregnant ewes in an advanced clinical stage of the disease were included in the study. From each fetus, amniotic fluid, brain, spleen, ileo-cecal valve and retropharyngeal lymph node samples were collected and analyzed using Western blotting and PMCA. Although all samples were negative using Western blotting, PrPres was detected after *in vitro* amplification. Our results represent the first time the biochemical detection of prions in fetal tissues, suggesting that the *in utero* transmission of scrapie in natural infected sheep might be possible.

## Introduction

Transmissible spongiform encephalopathies (TSEs) are fatal neurodegenerative diseases that affect both humans and animals. TSEs are characterized by long incubation periods, brain vacuolation and the accumulation of an abnormal isoform (PrPres) of a normal cellular protein (PrP^c^), mainly in nervous tissues. This abnormal protein is considered the only reliable biochemical disease marker [1]. Currently, most TSE diagnostic techniques are based on the immunodetection of PrPres in extracts or tissue sections of the central nervous system (CNS), mainly by Western blotting (WB), immunohistochemistry (IHC) and enzyme-linked immunosorbent assay (ELISA).

Scrapie is a TSE that affects sheep and goats. This disease is associated with a wide PrPres dissemination in many non-neural tissues, including the lymphoreticular system and the placenta [2], [3], and with high levels of transmissibility within susceptible populations, although the mechanisms of transmission are not completely understood. It is generally assumed that scrapie is transmitted by direct contact between animals or indirectly through the environment, where PrPres can exist for several years [4], [5]. In the case of scrapie in sheep, it is well known that the delivery period and the placenta play a key role in the transmission. Placentas from susceptible infected animals may harbor a high amount of PrPres and high levels of infectivity [6] when the fetus presents a susceptible PrP genotype [7], [8], [9].

In contrast, *in utero* transmission has never been clearly demonstrated. Limited studies have not detected infectivity by bioassay [10] or PrPres by conventional immunoassay of fetuses and fetal fluids [7], [8], [11]–[13]. To our knowledge, only two experimental studies suggest that *in utero* infection may occur [14], [15].

Recently, the development of highly sensitive methodologies has allowed the detection of PrPres in samples from infected scrapie animals that are undetectable by traditional assays. Using the protein misfolding cyclic amplification (PMCA) technique, PrPres has been detected in milk, feces, urine, saliva and blood [16]. PMCA involves the *in vitro* amplification of PrPres using a normal brain homogenate as the source of PrP^c^, leading to a several million-fold increase in sensitivity compared to standard WB assays [17]. The main goal of the present study was to attempt detection of PrPres in fetal tissues and their amniotic fluid from scrapie infected ewes using the PMCA technique.

## Results

### Detection of PrPres in dams by immunohistochemistry

**Figure 1:**
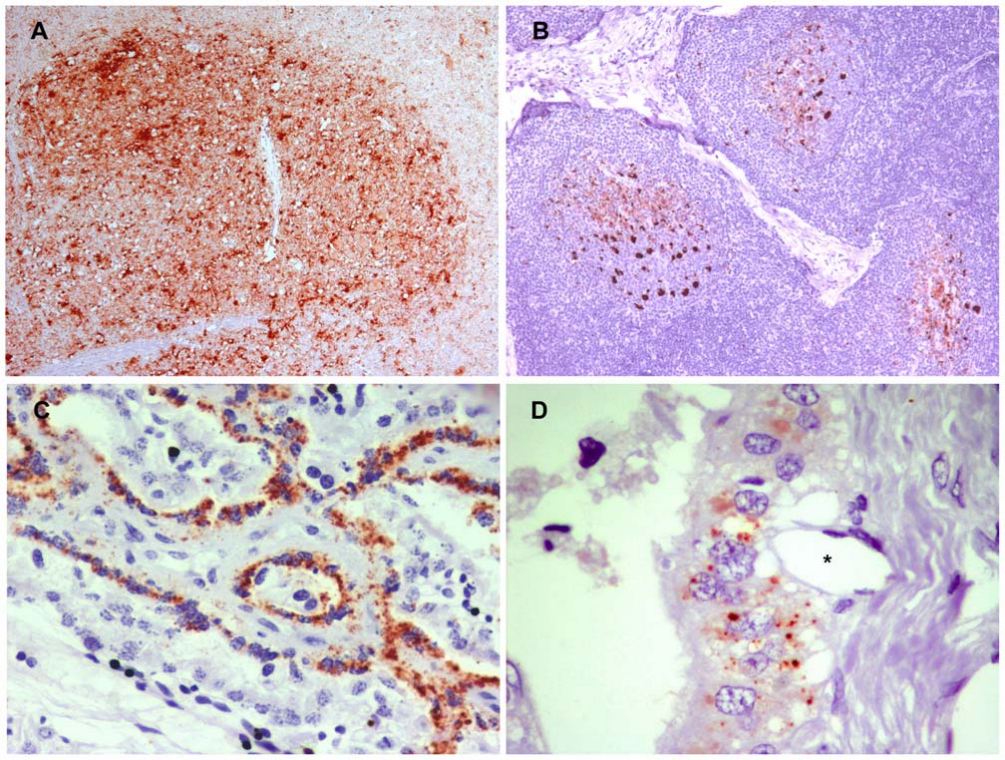
IHC detection of PrPres deposition in scrapie-infected dams. A wide PrP^Sc^ distribution was observed in the 3 scrapie-infected dams. A high intensity of immunolabeling in an obex (A), a retropharyngeal lymph node (B) and a placentome at the level of feto-maternal interface (C), is shown. In this image (D), the proximity between trophoblast cells presenting PrP^Sc^ deposits and a fetal vessel (*) can be observed.

A wide PrPres distribution was observed in the 3 ewes (Fig. 1). In the CNS, the following areas showed detectable PrPres levels: the cervical, thoracic and lumbar spinal cord; obex; pons; cerebellum; midbrain; diencephalon; pituitary; rhinencephalon; basal ganglia; olfactory bulb; amygdale; optic chiasm; eye; and the occipital, parietal and frontal cortex. All obex were scored as +++ (Fig. 1A). Regarding the lymphoreticular system (LRS), all tissues examined were scored as ++ or +++ (Fig. 1B). In the case of the palatine tonsil, retropharyngeal lymph node, spleen and lymphoid tissue associated with the ileocecal valve, more than 50% of the lymphoid follicles per section presented PrPres. In the case of control lamb tissues, no PrPres was detected.

All the placentomes examined were PrPres positive. PrPres deposition was multifocal along the transversal section of the placentome and was located mainly at the level of the feto-maternal interface. PrPres deposits were observed in endometrial caruncular cells, trophoblast cells and hybrid syncytial plaques (Fig. 1C, D).

All samples analyzed from the control lamb were negative using IHC analysis.

### Detection of PrPres in fetal tissues using Western blotting

The brain, spleen and amniotic fluid of all fetuses were negative for PrPres using WB analysis.

### Detection of PrPres in fetal tissues after serial automated PMCA (saPMCA)

**Figure 2:**
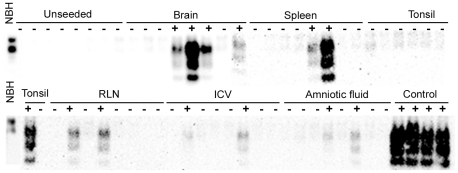
WB detection of PrPres in tissues from fetus 1 after saPMCA. After the 4th round of saPMCA, at least one of the eight aliquots analyzed from each tissue showed PrP^Sc^ amplification. None of the unseeded negative controls showed any detectable signal. NBH: Normal brain homogenate without pK treatment. RLN: retropharyngeal lymph node. ICV: ileocecal valve.

PrPres was detected in all fetuses after *in vitro* amplification (Table 1). Fetus 1 was analyzed first. In this case, samples were evaluated using WB after the 4th round of amplification, and those tubes that showed PrPres amplification were analyzed at rounds 3, 2 and 1. After the 1st and 2nd round of 96 cycles of saPMCA, none of the samples showed any detectable signal. After the 3rd round, samples from the brain and the spleen (2 and 1 tubes, respectively) showed a signal corresponding to amplified PrPres. After a 4th round, a PrPres signal was observed in samples of each kind (at least 1 tube of each tissue; Fig. 2). PrPres from a positive control was successfully amplified through each round, whereas none of the unseeded negative controls showed any detectable signal.

**Table 1:** PrPres amplification in fetal tissues after 3–4 rounds of saPMCA.

Fetus	Fetal genotype	Ewe	Month of gestation	PMCA Round	Brain	Spleen	RLN	ICV	Amniotic Fluid
1	ARQ/ARQ	A	5	3rd	2	1	0	0	0
				4th	4	2	2	2	2
2*	ARQ/AHQ	B	4	4th	3	0	4	2	1
3*	ARQ/ARQ	B	4	4th	3	2	1	0	1
4	ARQ/ARQ	B	4	3rd	1	2	0	0	0
5	ARQ/ARQ	C	3	3rd	1	0	NT	NT	NT
				4th	NT	NT	1	1	4
6	ARQ/ARQ	C	3	4th	4	5	1	3	4

Number of positive aliquots for each sample analyzed (from a total of 8 aliquots).

RLN: retropharyngeal lymph node. ICV: ileocecal valve. NT: not tested.

*Fetuses 2 and 3 shared the same uterine horn.

Samples from the other 5 fetuses were evaluated using WB after a 3rd and/or 4th round of amplification. As in the case of fetus 1, the PrPres signal was observed in all samples (although not in all fetuses). In these assays, positive controls were not included in the saPMCA to prevent any source of contamination inside the sonicator.

Samples from a negative lamb were analyzed to control contamination during sample collection and initial processing. After 4 rounds of amplification, no PrPres signal was observed in any tissue sample.

## Discussion

Scrapie is associated with high levels of transmissibility within susceptible populations. Although it is generally accepted that the natural spread of classical scrapie occurs mainly horizontally [5], [18], it is also known that there is an increased risk of developing the disease in offspring from scrapie-infected ewes [19]. A recent study has shown that this increased risk results not only from genetic susceptibility but also because of the potential for the infection to be transmitted from the dam to her offspring [20].

Until now, experimental studies have indicated that maternal transmission occurs in the immediate postpartum period via milk or contact with maternal contaminated tissues [18], [21]–[23].Our results suggest that *in utero* transmission can also be possible in natural scrapie-infected ewes. A previous study showed a vertical transmission in a transgenic mouse line expressing bovine PrP experimentally infected by intracerebral administration of BSE prions [15]. In this study, blood was proposed as the vehicle for prion spread due to its ability to retain prion infectivity [24]. However, there are differences with respect to the architectural anatomies of mouse and ruminant placentation. In the mouse placenta (hemotrichorial), all maternal tissue layers are removed such that 3 trophoblast layers plus a fenestrated endothelium separate the maternal and fetal blood. In the ruminant placenta (synepitheliochorial), all maternal and fetal tissue layers are present, although the uterine epithelium is greatly modified by migration and fusion of the fetal binucleate trophoblast cells with uterine epithelial cells. In this placenta type, contact between maternal blood and trophoectoderm occurs mainly in the hemophagous zones at the base of the fetal villi. In these zones, trophoblast cells phagocytose maternal erythrocytes (as a source of fetal iron), leukocytes and degenerated cells [25], [26].

As it has been previously reported, we have observed that in placentomes, PrPres accumulates mainly in trophoblast and derivative syncytial cells [7], [27]. However, it is unknown how trophoblast cells acquire PrPres. One possible way could be through phagocytosis of infected maternal blood in the hemophagous zones [28]. PrPres could also be transcytosed in vesicular structures across the fetal layers, forming complexes with other proteins, such as ferritin, as has been described for the intestinal epithelial barrier [29], [30]. This mechanism could be favored by the presence of many fetal capillaries in an “intraepithelial” position in the trophoblast, thus reducing the placenta barrier [31] (Fig. 1D). From fetal capillaries of the cotyledon, blood carrying PrPres could reach the fetus by the umbilical cord. PrP^c^ that is necessary for prion replication [32] is expressed in ruminant embryos starting day 4 post-fertilization [33]. Our results suggest that prions are present at higher levels in the brain and the spleen of fetuses than in other tissues (because they were detected by fewer PMCA cycles). A high concentration of PrP^c^ in the brain and the accumulation of blood in the spleen could explain these results.

Previous studies have failed to detect PrPres in fetal tissues from natural scrapie-infected ewes. However, in these studies, PrPres detection is based on a conventional immunoassay [7], [8], [11]–[13]. In the current study, the enhanced sensitivity of saPMCA allowed us to detect amplifiable prions in fetal tissues from sheep with clinical scrapie that presented a widespread PrPres deposition in the CNS and lymphoid tissue. The use of the tg338 mouse line as a substrate, which showed a higher sensitivity than other mouse lines tested previously (data not shown), could also have contributed to the sensitivity of the technique. The failure to detect PrPres in fetal tissues with conventional assays suggests that prions are present at very low levels. Previous studies have also amplified prions using saPMCA in tissues, secretions or excretions that tested negative when conventional assays were used [16], [24], [34], [35]. saPMCA is a highly sensitive technique that amplifies minute quantities of PrPres [17]. However, it has been suggested that there is a risk of obtaining false positive results when multiple rounds of PMCA are performed, due either to contamination or spontaneous generation [34]. Some authors [36] have shown that *de novo* generation of prions does not occur under standard PMCA conditions and that only when these conditions are modified can prions be generated after 9 and 10 rounds of PMCA (in the case of hamsters and mice samples, respectively). Using standard PMCA conditions, our results show that prions can be detected in the fetal brain and spleen after only three rounds of amplification. Although we cannot discard completely a cross-contamination, specific precautions (described in the material and methods) were taken to avoid it during sample collection at necropsy and saPMCA procedure.

Our results represent the first time that prions have been biochemically detected in fetal tissues, suggesting that *in utero* transmission of scrapie could be possible. This vertical transmission could result from the large accumulation of maternal blood in the placenta and subsequent feto-maternal interchange.

## Materials and Methods

### Ethics Statement

This study was carried out in strict accordance with the recommendations for the care and use of experimental animals of the University of Zaragoza, in accordance with law (R.D. 1201/2005). The protocol was approved by its Committee on the Ethics of Animal Experiments (Permit Number: PI02/08).

### Animals and samples

Three pregnant *Rasa aragonesa* sheep (A, B and C; Table 1) were selected from 3 different scrapie outbreaks detected in the framework of the scrapie surveillance program. Animals were selected on the basis of pregnancy, presenting an ARQ/ARQ genotype, having a positive rectal biopsy and presenting severe clinical signs. The signs observed were in accordance with previous clinical characterizations of scrapie in this breed [37].

When clinical signs progressed to terminal, ewes were euthanized by intravenous injections of sodium pentobarbital followed by exsanguination. From each ewe, several neural and lymphoreticular tissues and placentomes were sampled for PrPres detection using IHC and amniotic fluid and fetuses were collected for WB and saPMCA. At necropsy, the placenta was removed first, placed in a clean area and processed with different materials to avoid contamination from the infected dam. The amniotic fluid sample was first collected with a sterile syringe by making a small incision to separate the placental layers. After enlarging the incision, the fetus was carefully separated from the placenta and immediately frozen at −80°C in an individual plastic bag until sampled in a laminar flow cabinet in a different laboratory. From each ewe, the following tissues were collected and immersed in 10% buffered formalin: the spinal cord; cerebrum; cerebellum; palatine tonsils; spleen; ileocecal valve; and the retropharyngeal, mediastinal, mesenteric, submandibular, prescapular, iliac, precrural, popliteal and mammary lymph nodes. Five placentomes were collected from each fetus.

To control for contamination during sample collection, a 2-month-old ARQ/ARQ lamb from a negative flock was euthanised and sampled after the last ewe (ewe C; the day after). In this case, only the spleen and brain were collected in the necropsy room. One section of each tissue was frozen at −80°C until used for saPMCA, and another section was immersed in 10% buffered formalin for PrPres detection using IHC. All animals included in the present study were euthanised and sampled in the same necropsy room at the University of Zaragoza and under the same conditions. After each necropsy, all material and necropsy tables were cleaned with sodium hypochlorite and decontaminated with 1 M NaOH overnight.

A total of 6 fetuses were included in the present study (Table 1). Fetuses were sampled in a laminar flow cabinet at CIC bioGUNE laboratory. From each fetus, samples from the brain, spleen, ileocecal valve and retropharyngeal lymph node were collected. A palatine tonsil from fetus 6 was also collected. The materials used for sample collection was decontaminated between fetuses by immersion in 1 M NaOH.

### Immunohistochemistry

Samples from the ewes and the control lamb were analyzed using IHC to detect PrPres and assess its distribution. Formalin-fixed samples were trimmed and processed according to standard histopathological procedures. The IHC protocol for PrPres detection was performed as previously described [38]. Briefly, this included immersion in 98% formic acid for 15 min followed by proteinase K (F. Hoffmann La Roche Ltd, Switzerland; 4 µg/ml) treatment for 15 min at 37° and hydrated autoclaving. Slides were stained with an automated autostainer (Dako Denmark A/S, Denmark). Anti-prion mAb L42 (R Biopharm Ltd., Germany; 1∶500) was used as a primary antibody, followed by the application of EnVision (Dako Denmark A/S, Denmark) as a visualization system and diaminobenzidine (Dako Denmark A/S, Denmark) as the chromogen. Sections were washed in distilled water and counterstained with hematoxylin (Dako Denmark A/S, Denmark).

The PrPres signal was subjectively scored based on the extent of immunostaining [39], [40]. In the case of the medulla oblongata, + was characterized by the accumulation of PrPres in the dorsal motor nucleus of the vagus nerve, ++ was characterized by accumulation of PrPres in the dorsal motor nucleus of the vagus nerve and the adjacent nuclei, and +++ was characterized by widespread PrPres in the whole section. In other areas of the CNS, only the presence and absence of PrPres were assessed. In the case of lymphoid tissue, + was characterized by <10% of the lymphoid follicles with PrPres deposits, ++ was defined as 10 to 50% of the lymphoid follicles and +++ as >50% of the lymphoid follicles.

### Western blotting

From each fetus, CNS, spleen and amniotic fluid were analyzed using WB with Prionics®-Check Western Small Ruminant according to the manufacturer's instruction with the exception of the P4 mAb (R Biopharm Ltd., Germany; 1∶5000).

### saPMCA

Amniotic fluid and fetal tissues were analyzed by saPMCA as described previously [41]. Briefly, brains from perfused (5 mM EDTA in PBS) tg338 mice over-expressing the VRQ allele of ovine PrP [42] were used as the substrate for *in vitro* prion conversion and as unseeded negative controls. Mouse brain homogenates (10% w/v) were prepared in a conversion buffer (PBS containing 150 mM NaCl and 1% Triton X-100 with the addition of Complete Protease Inhibitors; Roche Pharmaceuticals, Indianapolis, IN). Fetal tissue samples were homogenized at a concentration of 10% (w/v) in GIBCO® PBS (calcium and magnesium free) with Complete Protease Inhibitors. Amniotic fluid was used without dilution in PBS. In 0.2 ml PCR tubes, 5 µl of fetal tissue homogenate or amniotic fluid was added to 50 µl of mice brain homogenate (substrate). For each sample to be tested, 8 aliquots were analyzed. Tubes were place on an adaptor on the plate holder of a microsonicator (Misonix, USA, model S3000MP sonicator). Unseeded substrates were used as negative controls. Substrates seeded with scrapie-infected brains were used as positive controls. Successive rounds of saPMCA consisting of 24 hours of cyclic amplification (incubation/sonication cycles) at 37°C were performed for amplification. Incubations were done in water without shaking. After each round, 10 µl of the amplified sample was diluted into 50 µl of fresh substrate for serial amplification.

To detect the amplified product, samples were evaluated using WB. One volume of amplified product was mixed with one volume of a digestion buffer (PBS, 2% Tween and 2% NP-40) and digested with proteinase K (85 µg/µl) for 60 min at 42°C with shaking (450 rpm). Digestion was stopped by adding 10 µl SDS Nu-PAGE loading buffer to all samples and incubating at 100°C for 10 min. Proteins were resolved by SDS-PAGE (12% gel, Invitrogen life) and transferred onto nitrocellulose membrane (GE Healthcare Whatman). Membranes were incubated for 1 h with P4 mAb (R Biopharm Ltd., Germany) diluted 1∶5000 in PBS with 0.05% Tween-20 and 1% milk. The secondary antibody was goat anti-mouse at 1∶3000 dilution in PBS 0.05% Tween-20 with 1% milk. After washing, immunoreactivity was detected using an enhanced chemiluminescent substrate (Thermo Scientific SuperSignal West Pico Chemiluminescent Substrate West Pico). Signals obtained by Western blotting were analyzed using FluorChem Q system (Alpha Innotech), and the data using AlphaView Q software.

Tissues from the control lamb were analyzed using saPMCA under the same conditions as the fetal tissues.

## References

[1] Prusiner SB (1998). Prions.. Proc Natl Acad Sci U S A.

[2] Sigurdson CJ (2008). A prion disease of cervids: chronic wasting disease.. Vet Res.

[3] Jeffrey M, Gonzalez L (2007). Classical sheep transmissible spongiform encephalopathies: pathogenesis, pathological phenotypes and clinical disease.. Neuropathol Appl Neurobiol.

[4] Miller MW, Williams ES (2003). Prion disease: horizontal prion transmission in mule deer.. Nature.

[5] Hoinville LJ (1996). A review of the epidemiology of scrapie in sheep.. Rev Sci Tech.

[6] Race R, Jenny A, Sutton D (1998). Scrapie infectivity and proteinase K-resistant prion protein in sheep placenta, brain, spleen, and lymph node: implications for transmission and antemortem diagnosis.. J Infect Dis.

[7] Andreoletti O, Lacroux C, Chabert A, Monnereau L, Tabouret G (2002). PrP(Sc) accumulation in placentas of ewes exposed to natural scrapie: influence of foetal PrP genotype and effect on ewe-to-lamb transmission.. J Gen Virol.

[8] Tuo W, O'Rourke K, Zhuang D, Cheevers W, Spraker T (2002). Pregnancy status and fetal prion genetics determine PrPsc accumulation in placentomes of scrapie infected shhep.. PNAS.

[9] Alverson J, O'Rourke KI, Baszler TV (2006). PrPSc accumulation in fetal cotyledons of scrapie-resistant lambs is influenced by fetus location in the uterus.. J Gen Virol.

[10] Hadlow WJ, Kennedy RC, Race RE (1982). Natural infection of Suffolk sheep with scrapie virus.. J Infect Dis.

[11] Ersdal C, Ulvund MJ, Espenes A, Benestad SL, Sarradin P (2005). Mapping PrPSc propagation in experimental and natural scrapie in sheep with different PrP genotypes.. Vet Pathol.

[12] Caplazi P, O'Rourke K, Wolf C, Shaw D, Baszler TV (2004). Biology of PrPsc accumulation in two natural scrapie-infected sheep flocks.. J Vet Diagn Invest.

[13] Onodera T, Ikeda T, Muramatsu Y, Shinagawa M (1993). Isolation of scrapie agent from the placenta of sheep with natural scrapie in Japan.. Microbiol Immunol.

[14] Hourrigan JL (1990). Experimentally induced bovine spongiform encephalopathy in cattle in Mission, Tex, and the control of scrapie.. J Am Vet Med Assoc.

[15] Castilla J, Brun A, Diaz-San Segundo F, Salguero FJ, Gutierrez-Adan A (2005). Vertical transmission of bovine spongiform encephalopathy prions evaluated in a transgenic mouse model.. J Virol.

[16] Gough KC, Maddison (2010). BC Prion transmission: prion excretion and occurrence in the environment.. Prion.

[17] Saá P, Castilla J, Soto C (2006). Ultra-efficient replication of infectious prions by automated protein misfolding cyclic amplification.. J Biol Chem.

[18] Wrathall AE, Holyoak GR, Parsonson IM, Simmons HA (2008). Risks of transmitting ruminant spongiform encephalopathies (prion diseases) by semen and embryo transfer techniques.. Theriogenology.

[19] Dickinson AG, Stamp JT, Renwick CC (1974). Maternal and lateral transmission of scrapie in sheep.. J Comp Pathol.

[20] Hoinville LJ, Tongue SC, Wilesmith JW (2009). Evidence for maternal transmission of scrapie in naturally affected flocks.. Prev Vet Med.

[21] Lacroux C, Simon S, Benestad SL, Maillet S, Mathey J (2008). Prions in milk from ewes incubating natural scrapie.. PLoS Pathog.

[22] Konold T, Moore SJ, Bellworthy SJ, Simmons HA (2008). Evidence of scrapie transmission via milk.. BMC Vet Res.

[23] Maddison BC, Rees HC, Baker CA, Taema M, Bellworthy SJ (2010). Prions are secreted into the oral cavity in sheep with preclinical scrapie.. J Infect Dis.

[24] Castilla J, Saá P, Soto C (2005). Detection of prions in blood.. Nat Med.

[25] Myagkaya G, Schellens JPM (1981). Final stages of erythrophagocytosis in the sheep placenta.. Cell and Tissue Research.

[26] Wooding P, Burton G (2008). Comparative placentation..

[27] Lacroux C, Corbiere F, Tabouret G, Lugan S, Costes P (2007). Dynamics and genetics of PrPSc placental accumulation in sheep.. J Gen Virol.

[28] Thorne L, Terry LA (2008). In vitro amplification of PrPSc derived from the brain and blood of sheep infected with scrapie.. J Gen Virol.

[29] Mishra RS, Basu S, Gu Y, Luo X, Zou WQ (2004). Protease-resistant human prion protein and ferritin are cotransported across Caco-2 epithelial cells: implications for species barrier in prion uptake from the intestine.. J Neurosci.

[30] Sunkesula SR, Luo X, Das D, Singh A, Singh N (2010). Iron content of ferritin modulates its uptake by intestinal epithelium: implications for co-transport of prions.. Mol Brain.

[31] Leiser R, Krebs C, Klisch K, Ebert B, Dantzer V (1997). Fetal villosity and microvasculature of the bovine placentome in the second half of gestation.. J Anat.

[32] Brandner S, Isenmann S, Raeber A, Fischer M, Sailer A (1996). Normal host prion protein necessary for scrapie-induced neurotoxicity.. Nature.

[33] Peralta OA, Huckle WR, Eyestone WH (2010). Expression and knockdown of cellular prion protein (PrPC) in differentiating mouse embryonic stem cells.. Differentiation.

[34] Haley NJ, Seelig DM, Zabel MD, Telling GC, Hoover EA (2009). Detection of CWD prions in urine and saliva of deer by transgenic mouse bioassay.. PLoS One.

[35] Kruger D, Thomzig A, Lenz G, Kampf K, McBride P (2009). Faecal shedding, alimentary clearance and intestinal spread of prions in hamsters fed with scrapie.. Vet Res.

[36] Barria MA, Mukherjee A, Gonzalez-Romero D, Morales R, Soto C (2009). De novo generation of infectious prions in vitro produces a new disease phenotype.. PLoS Pathog.

[37] Vargas F, Bolea R, Monleon E, Acin C, Vargas A (2005). Clinical characterisation of natural scrapie in a native Spanish breed of sheep.. Vet Rec.

[38] Monleon E, Monzon M, Hortells P, Vargas A, Acin C (2004). Detection of PrPsc on lymphoid tissues from naturally affected scrapie animals: comparison of three visualization systems.. J Histochem Cytochem.

[39] Monleon E, Garza MC, Sarasa R, Alvarez-Rodriguez J, Bolea R (2011). An assessment of the efficiency of PrPsc detection in rectal mucosa and third-eyelid biopsies from animals infected with scrapie.. Vet Microbiol.

[40] Spraker TR, Gidlewski TL, Balachandran A, VerCauteren KC, Creekmore L (2006). Detection of PrP(CWD) in postmortem rectal lymphoid tissues in Rocky Mountain elk (Cervus elaphus nelsoni) infected with chronic wasting disease.. J Vet Diagn Invest.

[41] Castilla J, Saá P, Morales R, Abid K, Maundrell K (2006). Protein misfolding cyclic amplification for diagnosis and prion propagation studies.. Methods Enzymol.

[42] Vilotte JL, Soulier S, Essalmani R, Stinnakre MG, Vaiman D (2001). Markedly increased susceptibility to natural sheep scrapie of transgenic mice expressing ovine prp.. J Virol.

